# Quantitative Trait Loci for Interhemispheric Commissure Development and Social Behaviors in the BTBR T^+^ *tf/J* Mouse Model of Autism

**DOI:** 10.1371/journal.pone.0061829

**Published:** 2013-04-15

**Authors:** Dorothy M. Jones-Davis, Mu Yang, Eric Rider, Nathan C. Osbun, Gilberto J. da Gente, Jiang Li, Adam M. Katz, Michael D. Weber, Saunak Sen, Jacqueline Crawley, Elliott H. Sherr

**Affiliations:** 1 Department of Neurology, University of California San Francisco, San Francisco, California, United States of America; 2 Laboratory of Behavioral Neuroscience, Intramural Research Program, National Institute of Mental Health, National Institutes of Health, Bethesda, Maryland, United States of America; 3 Department of Epidemiology and Biostatistics, University of California San Francisco, San Francisco, California, United States of America; National Institutes of Health/NICHD, United States of America

## Abstract

**Background:**

Autism and Agenesis of the Corpus Callosum (AgCC) are interrelated behavioral and anatomic phenotypes whose genetic etiologies are incompletely understood. We used the BTBR T^+^ *tf/J* (BTBR) strain, exhibiting fully penetrant AgCC, a diminished hippocampal commissure, and abnormal behaviors that may have face validity to autism, to study the genetic basis of these disorders.

**Methods:**

We generated 410 progeny from an F2 intercross between the BTBR and C57BL/6J strains. The progeny were phenotyped for social behaviors (as juveniles and adults) and commisural morphology, and genotyped using 458 markers. Quantitative trait loci (QTL) were identified using genome scans; significant loci were fine-mapped, and the BTBR genome was sequenced and analyzed to identify candidate genes.

**Results:**

Six QTL meeting genome-wide significance for three autism-relevant behaviors in BTBR were identified on chromosomes 1, 3, 9, 10, 12, and X. Four novel QTL for commissural morphology on chromosomes 4, 6, and 12 were also identified. We identified a highly significant QTL (LOD score = 20.2) for callosal morphology on the distal end of chromosome 4.

**Conclusions:**

We identified several QTL and candidate genes for both autism-relevant traits and commissural morphology in the BTBR mouse. Twenty-nine candidate genes were associated with synaptic activity, axon guidance, and neural development. This is consistent with a role for these processes in modulating white matter tract development and aspects of autism-relevant behaviors in the BTBR mouse. Our findings reveal candidate genes in a mouse model that will inform future human and preclinical studies of autism and AgCC.

## Introduction

Autism spectrum disorders (ASD) are a heterogeneous group of neurodevelopmental disorders that are behaviorally categorized based on three diagnostic criteria: (1) deficits in social interactions, (2) impairments in communication, and (3) stereotyped and repetitive behaviors with restricted interests [1]. Although there is evidence for the contribution of environmental and epigenetic mechanisms [2], [3] to the development of ASD, findings from sibling and twin studies [4], [5], [6] indicate that genetics is a substantial etiologic contributor to ASD. SFARI gene (http://gene.sfari.org), a curated database of ASD candidate genes, lists over 250 genes found to be associated with ASD. These genes fall into many categories, from ion channels, kinases and signaling pathway molecules, to cell adhesion molecules and developmental genes, as well as genes with mechanisms of action yet to be elucidated.

One salient mechanism posited to contribute to the underlying pathology of ASD is aberrant long-range neuronal connectivity. This is supported by numerous MRI studies showing decreased fractional anisotropy (FA) in major white matter tracts in individuals with autism, including the cingulum, uncinate fasciculi, occipitotemporal tracts, and most consistently, in the corpus callosum. This suggests that deficiencies in interhemispheric connectivity due to white matter anomalies might underlie autism symptoms [7], [8], [9], [10], [11], [12]. Genetic association studies support the hypothesis that connectivity may be perturbed in ASD as well, as many genes that affect synapse formation and axon guidance have been implicated in the pathogenesis of the disorder [13], [14], [15].

Development of the corpus callosum includes midline patterning, the birth and specification of commissural neurons, and precise axonal pathfinding. Deficits in these processes can result in Agenesis of the Corpus Callosum (AgCC), which occurs in approximately 1∶4000 live births, and can lead to a range of deficits, including ASD [16], [17]. Although genetics contribute to both autism and AgCC, the disease burden explained by already identified genes in both disorders is small, indicating a more heterogeneous and complex set of etiologies.

Finding discrete causative genes for complex traits (including understanding their epistatic interactions) in humans has been challenging [18], [19]. Animal models provide complementary evidence to understand the biology and underlying causes of these complex disorders. An important advantage of animal models is our ability to perform invasive phenotyping, standardize the environment, and precisely control the genetic makeup of the study population. The BTBR T+tf/J (BTBR) inbred mouse strain exhibits deficits in behaviors that may have face validity for the three main diagnostic criteria for autism: (1) impaired social interactions, (2) unusual patterns of ultrasonic vocalizations, and (3) repetitive behaviors [20], [21], [22], [23]. Furthermore, the strain is distinguished by the absence of a corpus callosum, a smaller to absent hippocampal commissure, and a normal to enlarged anterior commissure [24], similar to anatomic changes observed in patients with AgCC [16]. Thus, the BTBR strain provides a unique preclinical rodent model for both autism and AgCC.

To investigate the molecular determinants of autism-relevant behaviors and commissure development in the BTBR mouse, we phenotyped both adult and juvenile social behaviors as well as commissural morphology, and then performed linkage analysis in 410 F2 intercross progeny (F1×F1) with the C57BL/6J strain that exhibits normal social behaviors and has fully formed forebrain commissures. We identified six novel loci meeting genome-wide significance for autism-relevant behaviors. We identified a strong linkage peak at the distal end of chromosome 4 for callosal morphology, as well as additional linkage peaks for hippocampal and anterior commissure size on chromosomes 6 and 12, respectively. Using deep sequencing of the BTBR genome, we identified candidate genes and variants for further investigation.

## Materials and Methods

### Ethics Statement

All behavioral procedures were conducted in strict compliance with the NIH Guidelines for the Care and Use of Laboratory Animals and approved by the National Institute of Mental Health Animal Care and Use Committee (Protocol Number: SGB-01-09). DNA testing (tail snips) and mouse perfusion procedures were conducted in strict compliance with the UCSF Guidelines for the Care and Use of Laboratory Animals and approved by the UCSF Institutional Animal Care and Use Committee (Protocol Number: AN084397-02). At both locations, all efforts were made to minimize any animal suffering.

### Assessment of Autism-relevant Behaviors in the BTBR Mouse Strain

C57BL/6J (B6) and BTBR T+tf/J (BTBR) mice breeding pairs were purchased from the Jackson Laboratory (Bar Harbor, ME) and bred at NIMH in Bethesda, Maryland. Subject mice were weaned at 21±1 days of age, then group housed by sex and strain in standard mouse cages containing 2–4 mice. Standard rodent chow and tap water were available *ad libitum*. In addition to standard bedding, a Nestlet square and a cardboard tube were provided in each cage. The colony room was maintained on a 12∶12 light/dark cycle, with lights on at 7∶00 AM. All experiments were conducted in the light phase, between 9∶00 AM and 5∶00 PM. A total of 410 F2 mice were generated for behavioral testing and QTL analysis over a period of two years. The first cohort of 204 mice was generated by crossing F1 males and females derived from BTBR female ×B6 male matings. The second cohort of 206 mice was generated by the reciprocal cross of F1 males and females derived from B6 female×BTBR male matings. This ensured equal representation of the BTBR and B6 X chromosomes in the final F2 cohort. The two cohorts were behaviorally tested over the same period of time. Both cohorts had an equal balance of contribution of the X chromosome from the parental strains.

#### Juvenile reciprocal social interaction

Juvenile reciprocal social interactions were tested on postnatal day 21, immediately before pups were weaned from the mother. The test was conducted in the Noldus PhenoTyper 3000 chamber (25 cm×25 cm×35 cm, Noldus, Leesburg, Virginia) as previously described [25], [26], [27], [28]. The floor of the arena was covered with a 0.5 cm layer of clean bedding. Each subject mouse was singly housed in a clean cage for one hour before the test. After this brief isolation period, the freely moving subject mouse and a freely moving age- and sex-matched B6 partner mouse were simultaneously placed in the arena and their interactions were videotaped for 10 min. Social interactions were scored by a highly trained observer, using the Noldus Observer 5.0 software. Identities of the F2 mice were coded throughout the test sessions and scoring. Parameters of social behaviors included nose-to-nose sniff (sniffing the nose and snout region of the partner), front approach (moving towards the partner from a distance, in a head-on manner), follow (walking straight behind the partner, keeping pace with the one ahead), nose-to-anogenital sniff (sniffing the anogenital region of the partner), and push-crawl (pushing the head underneath the partner’s body or squeezing between the wall/floor and the partner, and crawling over or under the partner’s body, combined as a single parameter). Non-social arena exploration (walking around the arena, rearing, or sniffing the wall) and bouts of self-grooming were scored as measures of exploratory activity and repetitive behavior, respectively. All behaviors were analyzed for frequency of occurrence, i.e. number of bouts.

#### Automated three-chambered social approach task

Social approach was assayed in our automated three-chambered apparatus (NIMH Research Services Branch, Bethesda, MD) using methods previously described [20], [25], [26], [27], [28], [29], [30], [31], [32], [33], between 8 and 12 weeks of age. The apparatus was a rectangular, three-chambered box made of clear polycarbonate. Retractable doorways built into the two dividing walls controlled access to the two side chambers. Number of entries and time spent in each of the three chambers were automatically detected by photocells embedded in the doorways and tallied by the software. The test began with a 10 min habituation session in the center chamber only, followed by a 10 min habituation to all three empty chambers. The subject was then briefly confined to the center chamber while the clean novel object (an inverted stainless steel wire pencil cup, Galaxy, Kitchen Plus, http://www.kitchen-plus.com) was placed in one of the side chambers. A novel mouse, previously habituated to the enclosure, was placed in an identical wire cup located in the other side chamber. A disposable plastic drinking cup containing a lead weight was placed on the top of each inverted wire pencil cup to prevent the subject from climbing on top. The side containing the novel object and the novel mouse alternated between the left and right chambers across subjects. After both stimuli were positioned, the two side doors were simultaneously lifted and the subject was allowed access to all three chambers for 10 min. Time spent in each chamber and entries into each chamber were automatically tallied. In addition, time spent sniffing the novel mouse and time spent sniffing the novel object were scored by human observers. Up to four subject mice were tested in the same room at the same time, using a high-throughput multi-unit arrangement of the 4 test chambers.

#### Repetitive self-grooming

Mice were scored for spontaneous grooming behaviors when placed individually in a clean, empty mouse cage without bedding, using methods previously described [20], [26]. Each mouse was given a 10 min habituation period in the empty cage and then rated for 10 min for cumulative time spent grooming all body regions. The test session was videotaped and scored later by two trained observers. Inter-rater reliability was >95%.

### Assessment of Commissural Morphology in the BTBR Mouse Strain

After behavioral testing, the F2 progeny were perfused with 4% PFA, and brains were stored in 1X phosphate buffered saline (PBS). Following perfusion, mouse brains were hemisected sagitally, and all myelin containing structures, including the corpus callosum, were stained with 0.2% buffered gold chloride solution followed by fixation for 5 minutes in 2.5% sodium thiosulfate. Stained hemisections were subsequently returned to 1X PBS for storage. Measurements of midsagittal corpus callosum length, corpus callosum area, hippocampal commissure length, hippocampal commissure area, and anterior commissure area were quantified using the ImageJ program (http://imagej.nih.gov/ij/).

### QTL Mapping for Autism-relevant Behaviors and AgCC in BTBR Mice

DNA samples from tail snips of the mice were purified using the Qiagen DNeasy Blood and Tissue kit (Qiagen; Valencia, CA) according to the manufacturers instructions. For the initial genome scan, SNPs were chosen using the Jackson Lab SNP tool (http://www.informatics.jax.org/javawi2/servlet/WIFetch?page=snpQF), and genotyping was conducted using the Sequenom Mass Array Analyzer 4 (Sequenom; San Diego, CA). In the initial genome scan, ten regions reached genome-wide significance. Based on this finding, we selected these ten regions for dense genotyping using the same methods as the initial scan.

### Statistical Analysis

We made histograms of all traits, and scatterplots of all pairs of traits as a diagnostic check. For traits that were measured in both cohorts (batches), we used cohort (batch) as an additive covariate in the QTL analysis, to adjust for any cohort (batch) effects. Genotype diagnostics were performed by comparing the genotype frequencies to the expected 1∶2∶1 ratio, and by examining the correlations between markers. Markers failing the quality checks were removed from consideration, and the genetic map was re-estimated.

One-dimensional genome scans were performed using the Haley-Knott (HK) algorithm with sex as covariate. If a trait was measured in both cohorts, we used cohort as a covariate in addition to sex. Genomewide statistical significance was determined using 1000 permutations. A LOD was considered significant when p<0.05, and was considered suggestive when 0.05<p<0.10. QTL confidence intervals were conducted using a Bayesian approach [34]. The QTL statistical analysis was conducted using the R programming language [35] and the R/qtl package [36]. The percent variances for each QTL were calculated in R/qtl by transformations of the LOD scores using the formula p = 1−10∧(− (2/n)*LOD). With 410 F2 animals, markers spaced approximately 10 cM, and using a LOD threshold of 3.39 for declaring significance, we have 80% power to detect QTL with additive effects exceeding 5.65% or more of the variance. Calculations were performed using R/qtlDesign [37].

### Whole Genome Sequencing of the BTBR T+tf/J Mouse Strain

Genome libraries were prepared following the standard Paired-End Sample preparation protocol provided by Illumina (http://www.illumina.com/support/sequencing/sequencing_kits/pe_dna_sample_prep_kit/documentation.ilmn). Briefly, 5 µg of genomic DNA was purified from mouse brain and sheared with the Covaris S220. To this fragmented treated DNA, the Illumina paired end adapters were ligated followed by ∼400 bp size selection through gel electrophoresis. The purified DNA was enriched by six cycles of PCR and the final product was purified using Agencourt Ampure SPRI beads. The libraries were then sequenced on the HiSeq 2000 with TruSeq v3 chemistry.

### Alignment and Variant Calling

Reads from two different Illumina runs were aligned to the July 2007 assembly of the mouse genome (mm9) using Novoalign (V2.07.17). A threshold value was set such that an alignment to a no repeat should have a phred scaled quality value higher than 30 (a statistical estimate of 0.1% that the read is incorrectly mapped). The resulting 357,291,496 aligned reads were sorted, indexed, and combined in one sequence alignment file (SAM) using SAMTools (http://samtools.sourceforge.net/) and 22,235,856 read duplicates (6% duplication rate) were removed using the Picard tool set (http://picard.sourceforge.net). Local realignment around identified indels was performed on the alignment file using the Genome Analysis ToolKit (GATK). The resulting base coverage analysis showed over 96% coverage over the reference genome with an average depth of 12 and over 99% coverage (90% with depth greater than 5) for the reference exome with an average depth of 16. Variant calling was performed using GATK’s Unified Genotyper with min_base_quality_score, stand_emit_conf, and stand_call_conf thresholds set to 10, 10, and 20 respectively giving us the largest possible subset for further analysis. The resulting VCF formatted call set contained 5,474,033 variants.

### Variant Annotation and Filtering

Custom software with a mySQL database backend was created in order to annotate, analyze, and store variant calls using a VCF formatted file as default input. After initial parsing of VCF file into a into our database schema structure, transcript annotation was done using the refGene mm9 build database from UCSC as reference. Variants were annotated based on location and distance from exon boundaries. Variants identified as having a non-synonymous change were further analyzed using Polyphen-2 (http://genetics.bwh.harvard.edu/pph2/) for predictive damage. All variants were also annotated against public BTBR, LP/J, and B6 call sets obtained from Sanger and CGD (http://cgd.jax.org/cgdsnpdb/utils/snp_data_report.php), which included the high quality mouse diversity array (http://cgd.jax.org/datasets/popgen/diversityarray/yang2011.shtml. The Sanger data was provided by the Mouse Genomes Project and Sanger Mouse Exomes Project groups at the Wellcome Trust Sanger Institute and can be obtained from: http://www.sanger.ac.uk/resources/mouse/genomes/. BTBR variants found to overlap with any public variant for any strain or repository mentioned previously are annotated as such in our database for quick query. As expected and as proof of process a high level of concordance was noted between our variants and variants from publicly available BTBR strain data. Our initial call set, now annotated and accessible in our database, is filtered with the criteria that variants are within identified QTL region, non-synonymous or within 2 base pairs from a splice position, and are not found in either BTBR or LP/J strain.

### Identification of Potential Gene Candidates

Following fine mapping, QTL meeting genome-wide significance of 5% were further analyzed to identify a list of gene candidates for future study. Sequencing the BTBR exome at 16X coverage allowed for evaluation of both novel and known variants within the identified QTL. Only variants within these regions that had an effect on protein coding or were within 2 base pairs of a splice position were identified, variants in regulatory regions (including 5′ and 3′ UTR in mRNA and promoter and enhancers) were not included in this analysis. A subset of those variants that differed from both the C57BL/6J parental strain, as well as the LP/J strain (which has a normal corpus callosum) were prioritized, while variants found to be the same in either were filtered using the criteria detailed above). Rationale for using the LP/J strain for this analysis was based on an assessment of genetic relatedness, which indicated that the LP/J was genetically very close to the BTBR strain, while genetically distant from the C57BL/6J strain. Yet the LP/J strain, similar to the C57BL/6J strain, also maintains a normal corpus callosum. The list was then further analyzed using the Mouse Genome Informatics (MGI) Genome Browser on Build 37 (http://gbrowse.informatics.jax.org/cgi-bin/gbrowse/mouse_current/), and the MGI Gene Expression Database (GXD - http://www.informatics.jax.org/expression.shtml). Resulting gene query was filtered to only include those involved in neurological behavior or within the nervous system, and a prioritized candidate list was generated for each locus. Final gene candidate priority was determined by multiple factors including: (1) Presence of a potentially pathogenic SNP, or indel in the BTBR strain within the gene, (2) distance of gene to QTL peak, (3) neuroanatomical distribution of gene (expression to include the mouse cerebral cortex), (4) temporal expression of gene (expression to include embryonic or early postnatal expression in mouse) and (5) prior known involvement of gene in neurological behavior, neural development, axonal guidance, cell adhesion, synaptic plasticity, absence, dysplasia or hypoplasia of the corpus callosum or other midline commissures, mental retardation, or ASD. All potentially pathogenic SNPs fulfilling these criteria were validated using Sanger sequencing. Following identification, all candidate genes with validated potentially pathogenic SNPs were then placed into four categories (developmental proteins, synaptic and receptor proteins, kinases, and immune proteins) based on a literature review assessing their indicated major function.

## Results

### Animal Model of Autism-relevant Behaviors: Behavioral Phenotype of the BTBR T^+^ *tf/J* Mouse Strain

**Figure 1:**
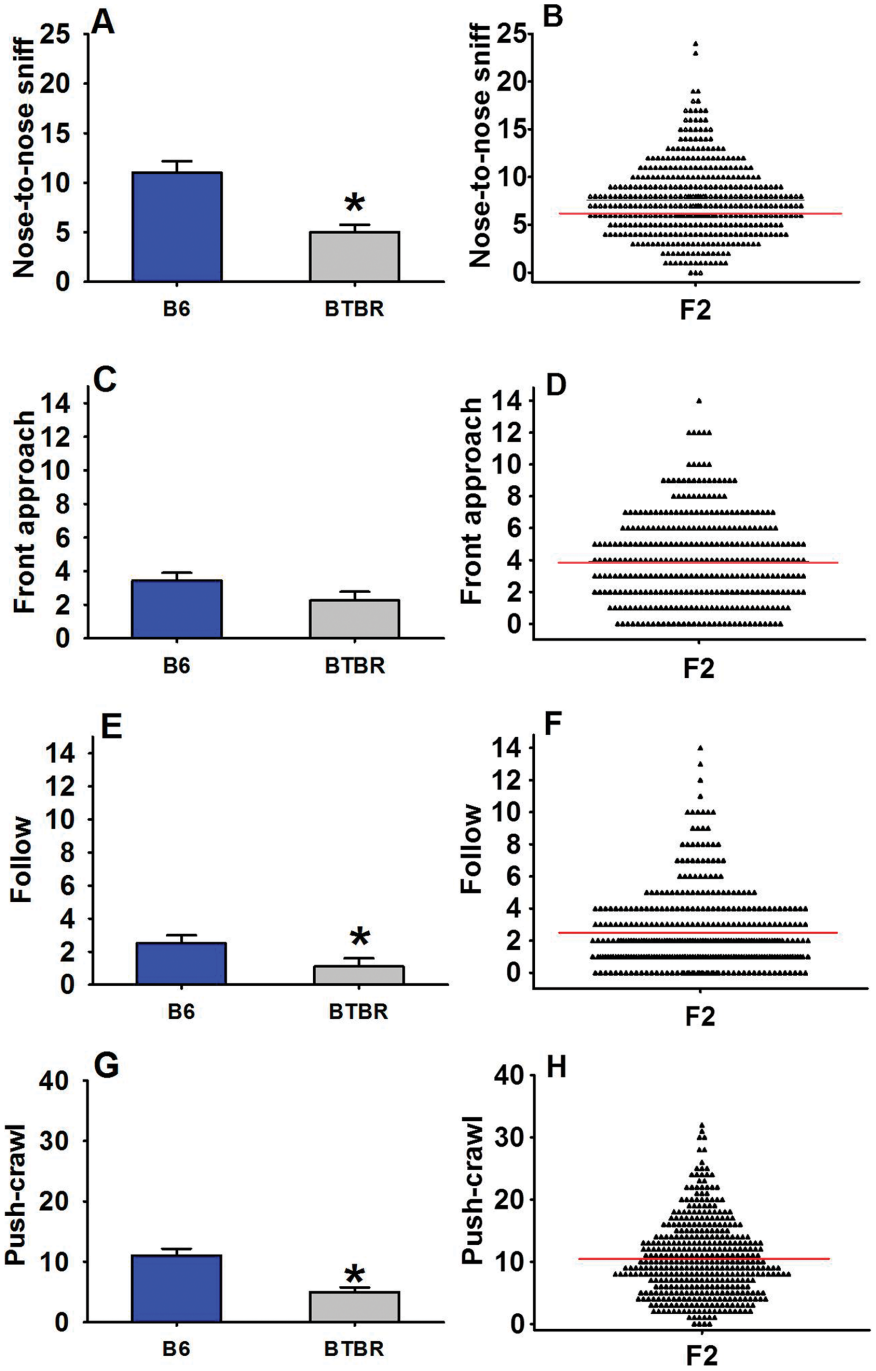
Juvenile social interactions in B6, BTBR, and F2 mice. (A) As compared to B6, BTBR exhibited significantly lower levels of nose-to-nose sniff, (E) follow, (G) push-crawl, (C) and a trend toward lower levels of front-approach. (B, F, H, D) In the present study, F2 subjects exhibited broad ranges of these behaviors. A, C, E, G display Mean ± SEM; bold red line in B, D, F & H indicates mean value. *B6 and BTBR data from A, E and G are adapted from Yang et* *al., 2009, European Journal of Neuroscience, Copyright (c) 2009, Blackwell Publishing, Ltd., with reuse and reprint permission via the Copyright Clearance Center (CCC).*

**Figure 2:**
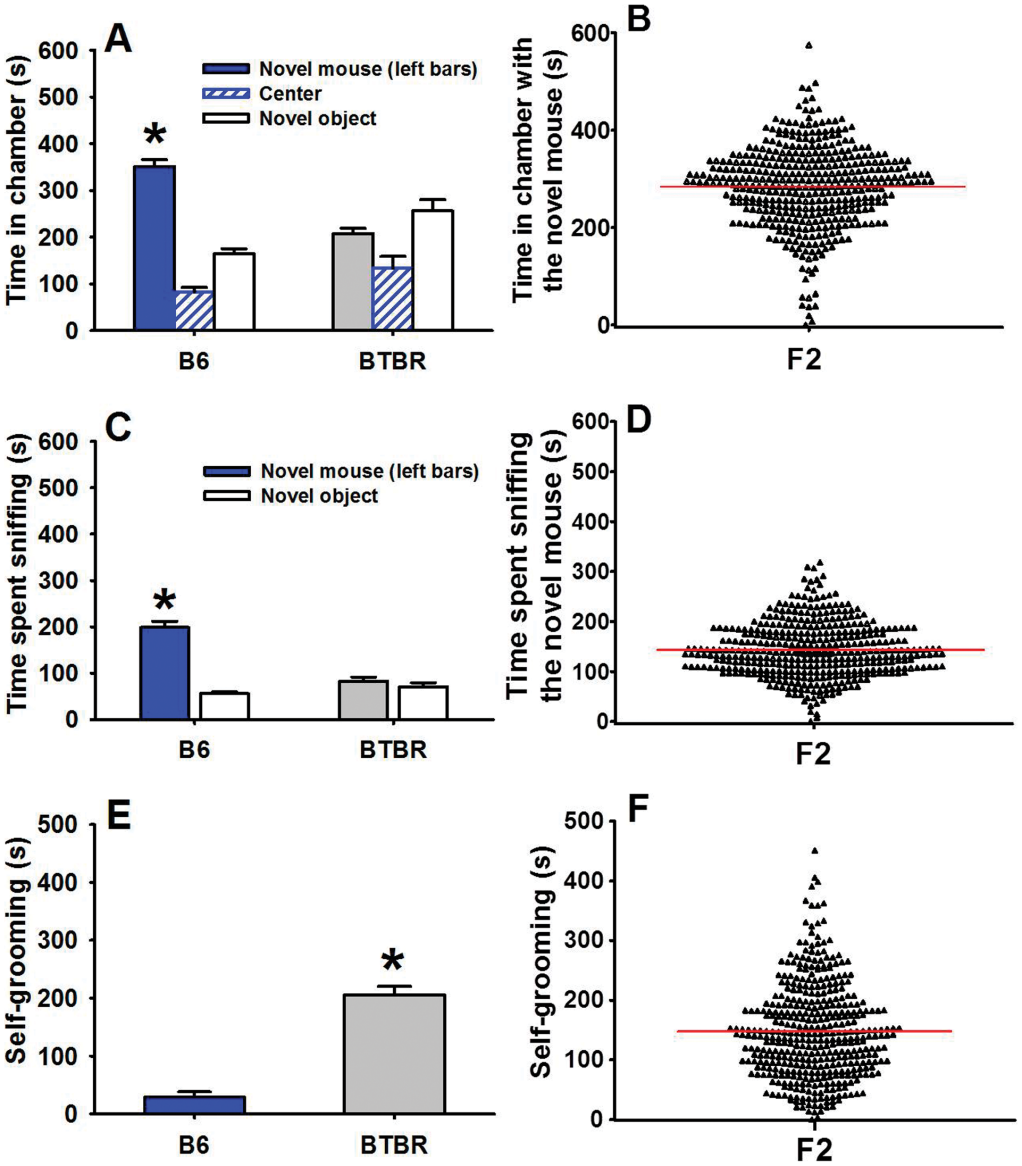
Adult social approach in B6, BTBR, and F2 mice. (A) B6 exhibited high sociability, spending significantly more time in the chamber containing the novel mouse than in the chamber containing the novel object, and (C) more time sniffing the novel mouse than the novel object. BTBR exhibited low sociability and did not show preference for the novel mouse. (E) As compared to B6, BTBR mice exhibited significantly higher levels of self-grooming in an empty cage. In the present study, F2 mice exhibited broad ranges of time spent in the novel mouse chamber (B), time sniffing the novel mouse (D), and self-grooming in an empty cage (F). The parental strains and F2 generation were tested using identical methods, by the same NIMH investigators in the Bethesda laboratory. The F2 scores shown in panel B and panel D represent the time spent in the chamber with the novel mouse Panel A and the time spent sniffing the novel mouse and time spent sniffing the novel object in Panel C, respectively. (A, C)*p<.05 for the comparison between novel mouse and novel object; (E)*p<.01 vs. B6. A, C, E display Mean ± SEM; bold red line in B, D, F indicates mean value. *B6 and BTBR data from A, C, and are adapted from Yang et* *al., 2009, European Journal of Neuroscience, Copyright (c) 2009, Blackwell Publishing, Ltd., with reuse and reprint permission via the Copyright Clearance Center (CCC).*

B6 and BTBR inbred mouse strains differ greatly in social and repetitive behaviors, relevant to the first and third diagnostic symptoms of autism. As compared to B6 (n = 11), BTBR (n = 12) mice display lower reciprocal social interaction as juveniles and adults, lower social approach behaviors, and high repetitive self-grooming [20], [27], [30] (Figures 1 and 2). In preparation for conducting this present study we tested F1 offspring generated by crossing (BTBR females X B6 males). Results showed that F1 juveniles scored intermediate between B6 and BTBR on most measures of social interaction behaviors, and adult F1 exhibited social deficits in the three-chamber test similar to BTBR (Figure S1). F2 juveniles (Figure 1) and adults (Figure 2) exhibited a broad distribution of behavioral phenotypes that were intermediate between B6 and BTBR.

### Animal Model of AgCC: Callosal Phenotype of the BTBR T^+^ *tf/J* Mouse Strain

**Figure 3:**
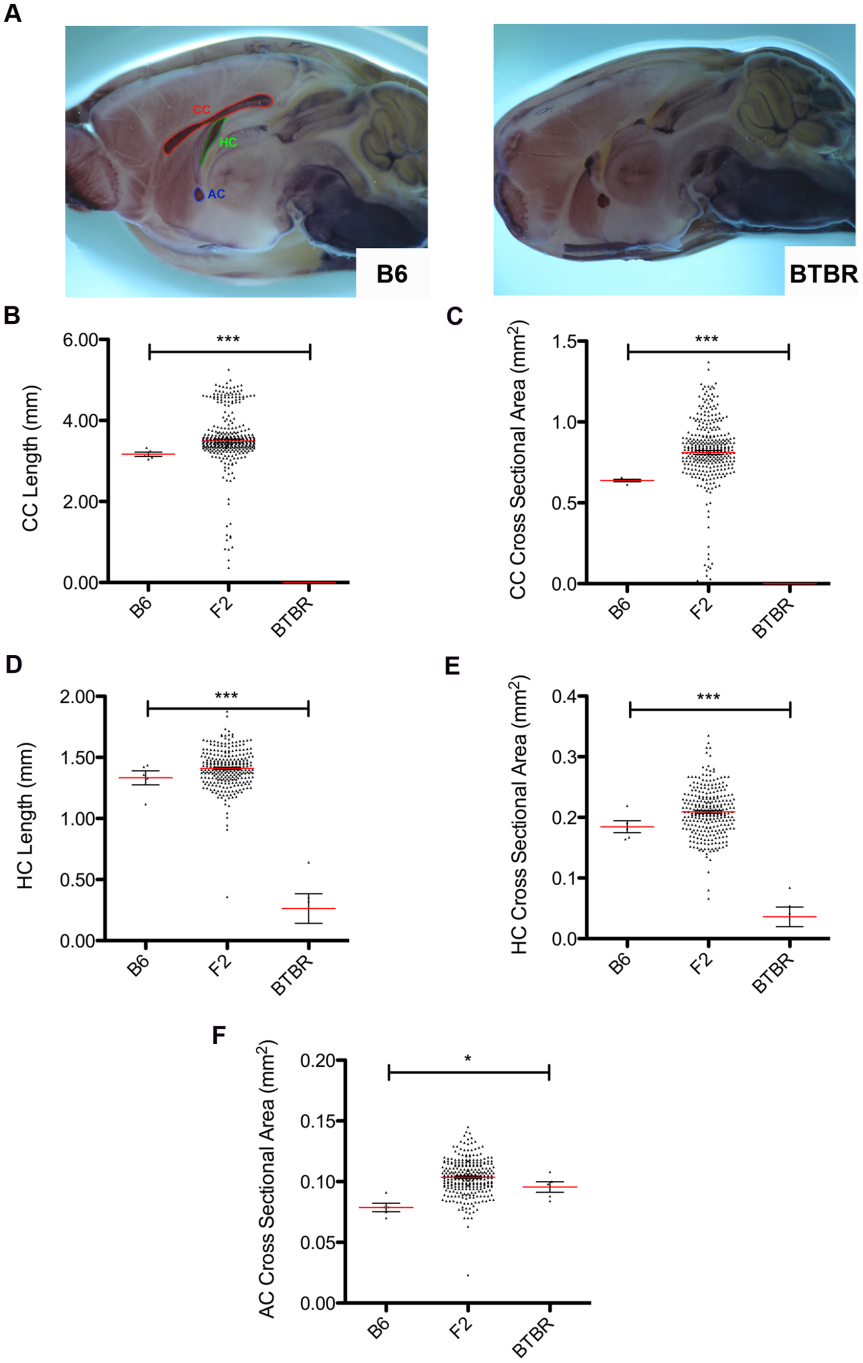
Callosal phenotype of the BTBR T^+^ *tf/J* mouse. (A) As compared to the C57BL6/J mouse strain, a midsaggital section of the BTBR T^+^ *tf/J* mouse brain displays deficits in the formation of commissural structures (CC – red, HC – green; AC – blue); (B, C) a complete loss of the corpus callosum (CC), a reduction in the (D) length and (E) area of the hippocampal commissure (HC), and (F) a slightly enlarged anterior commissure (AC). (B,–F) Graph of the distribution of (B) corpus callosum, and (D) hippocampal commissure lengths, and (F) anterior commissure areas of F2 intercrossed progeny, indicates a widespread distribution.

In 2003, Wahlsten et al. reported data on commissural morphology (corpus callosum (CC) area, hippocampal commissure (HC) area, and anterior commissure (AC) area) in 21 different mouse strains [24]. This was the first study to demonstrate complete callosal agenesis in the BTBR mouse strain. We confirmed this finding (Figure 3A, C) and also observed the reduced hippocampal commissure area (Figure 3A, E) previously noted in the strain. We also found a significant difference in the length of both the corpus callosum and hippocampal commissure in the strain (as measured in the mid-sagittal plane; Figure 3A, B, D; *Student’s t-test, p<0.001*). Interestingly, although the 2003 Wahlsten study did not find a significant difference in midsagittal anterior commissure area, an earlier study by Livy et al. [38] demonstrated an increased number of anterior commissure axons in acallosal mice. In the present study, we observed that the BTBR strain has a significantly larger anterior commissure than the B6 strain, as assessed by the midsagittal anterior commissure cross-sectional area (Figure 3A, F; *Mean ± SD*: BTBR: 0.0956±0.0100, B6∶0.0788±0.0080, *Student’s t-test, p<0.05*).

All B6 parental mice (n = 5) had normal corpus callosa, while the corpus callosum was absent from all BTBR mice (n = 5) (Figure 3A–C). Approximately 3% of F2 intercrossed mice demonstrated an absent corpus callosum, with the majority demonstrating an intermediate phenotype, suggesting an inheritance pattern more complex than single-gene Mendelian recessive (predicting 25% AgCC) (Figure 3B, C). Additionally, there was a secondary cluster of F2 intercrossed animals with commissures that were longer in the mid-sagittal plane than those found in B6 parental mice (Figure 3B, 3D). This distribution of commissural length within the F2 cohort is consistent with a complex inheritance pattern. A similar trend was noted for commissural area (3C, 3E, 3F).

### Identification of Quantitative Trait Loci for Autism-relevant Traits in the BTBR Mouse

**Figure 4:**
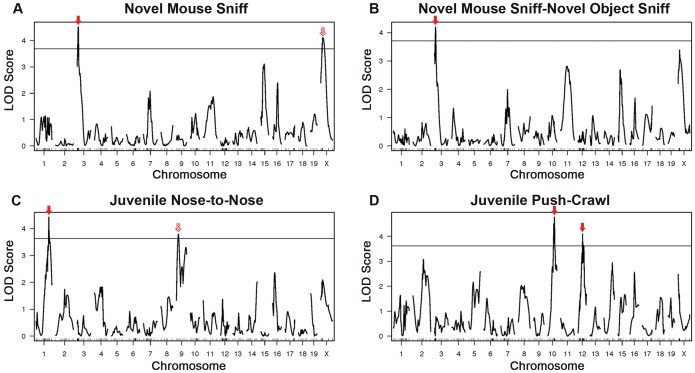
QTL for autism-relevant behaviors. For the related juvenile reciprocal social interaction trait parameters “novel mouse sniff” (the cumulative time spent sniffing the novel mouse) and “diff sniff” (the difference between seconds spent sniffing the novel mouse and seconds spent sniffing the novel object) (A, B), a significant QTL was found on chromosome 3 (LOD = 4.52). (B) An additional QTL was found on the X chromosome (LOD = 4.12) for the “novel mouse sniff” trait, but did not meet genome-wide significance for the trait “diff sniff.” (C) The trait “Juvenile Nose to Nose” displayed a QTL on chromosome 1 (LOD = 4.43) and chromosome 9 (LOD = 3.80). (D) The trait “Juvenile Push Crawl” displayed QTL on chromosome 10 (LOD = 4.77) and chromosome 12 (LOD = 4.09), respectively. Red arrows indicate statistically significant QTL; red hatched arrows indicate transgressive loci.

QTL mapping yielded a number of loci exceeding the 10% genome-wide permutation threshold (Table 1). Many QTL exceeded the 5% threshold, and were therefore prioritized as important loci for future study. Seven QTL for four autism-relevant behavioral traits, exceeding the 5% genome-wide permutation threshold, were identified (Figure 4 and Table 1). Two of the QTL, Novel Mouse Sniff (time spent sniffing the novel mouse) in the adult social approach task, and Diff Sniff (the difference between the time spent sniffing the novel mouse and the novel object), are related in nature, and as such, have overlapping peaks, which is expected, as they represent different parameters of a single behavioral trait. The Novel Mouse Sniff QTL has two peaks, one major peak on chromosome 3 with a LOD of 4.52 that was overlaps the Diff Sniff QTL (LOD = 4.21), and a suggestive secondary peak on the X chromosome with a maximum LOD of 4.12, that was transgressive (e.g. the B6 allele associated with a decrease in sniff time), and did not meet genome-wide significance as a QTL for “diff sniff.” After performing linear regression on all of the autism-relevant traits analyzing sex as a covariate, no sex effects could be identified.

**Table 1:** List of QTL for Autism-Relevant Behaviors and Callosal Phenotypes.

QTL	Chr	Peak (bp)	CI (bp)	LOD	P value	% Variance
**Novel Mouse Sniff**	3	18,932,880	5,370,727–32,421,916	4.52	0.00397*	8.6%
	X	61,160,361	45,996,141–132,120,932	4.12	0.01190*	
**Diff Sniff**	3	18,091,440	5,370,727–32,421,916	4.21	0.0109*	8.0%
	X	50,519,512	33,548,080–132,120,932	3.39	0.0893	
**Juvenile Nose to Nose**	1	174,420,463	157,761,451–193,462,277	4.43	0.0109*	6.8%
	9	35,057,843–44,008,819#	25,697,557–120,065,853	3.80	0.0347*	
**Juvenile Push Crawl**	10	101,426,263	75,175,591–113,178,567	4.77	0.00298*	10.3%
	12	35,057,843–44,008,819#	53,911,049–98,047,413	4.09	0.01687*	
**CC Length**	4	147,487,273	131,622,455–155,284,926	20.21	<0.001*	15.4%
	15	78,240,804–97,580,284#	59,691,746–97,580,284	3.45	0.0873	
**CC Area**	4	131,622,455–140,401,003#	122,811,425–147,768,403	10.9	<0.001*	10.5%
**HC Length**	4	131,622,455–140,401,003#	122,811,425–149,683,530	5.27	<0.001*	14.1%
	6	103,254,671	92,537,118–111,982,681	5.36	<0.001*	
**AC Area**	4	145,114,432	131,622,455–155,284,926	4.48	0.006944*	13.1%
	12	30,474,821	5,967,934–43,749,565	6.11	0.000992*	

* 5% genomewide significance (p≤0.05).

#In these cases, the peak coincided with an imputed marker, preventing us from determining its exact bp location. The location of the peak is therefore presented as the range between the two closest markers before and after the peak, respectively.

For the Juvenile Nose to Nose Sniff behavior in the juvenile reciprocal social interaction test, two QTL were found, one on chromosome 1 (LOD = 4.43), and a second on chromosome 9, that was transgressive (LOD = 3.80). The final autism-relevant behavior with significant linkage peaks was “Juvenile Push Crawl”, with one peak on chromosome 10 (LOD = 4.77) and a second peak on chromosome 12 (LOD = 4.09). We did not identify a statistically significant QTL for repetitive behaviors.

### Identification of Quantitative Trait Loci for Commissural Traits in the BTBR Mouse

**Figure 5:**
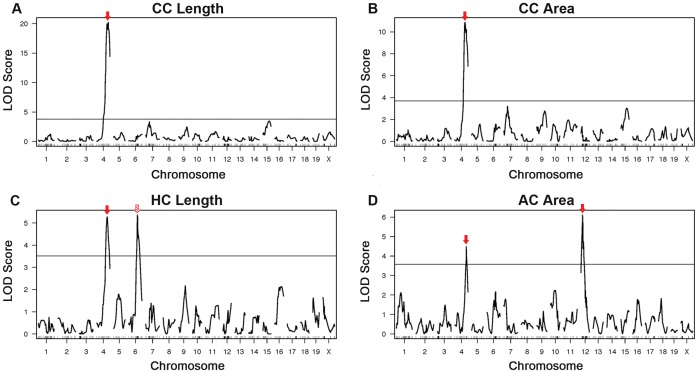
QTL for commissural morphology. For the trait “corpus callosum length” (A), a significant QTL was found on chromosome 4 (LOD = 20.21). (B) Interestingly, a different QTL, also located on chromosome 4, was found for the “corpus callosum area” trait (LOD = 10.9), and (C) was also noted as a secondary locus for the trait “hippocampal commissure length” (LOD = 5.27), whereas the primary locus for this trait was found on chromosome 6 (LOD = 5.36). (D) The trait “anterior commissure area” displayed a novel QTL on chromosome 12 (LOD = 6.11), and additionally, shared the same locus on chromosome 4 (LOD = 4.48) as the trait “corpus callosum length.” Red arrows indicate statistically significant QTL; red hatched arrows indicate transgressive loci.

Six QTL that linked to four commissural traits met a genome-wide significance of 5% (Figure 5 and Table 1). Notably, all significant QTL for commissural traits had high LOD scores (LOD >3), and all peaks met a significance level of p<0.01 (Table 1). In addition to peaks on chromosome 4, the traits “HC Length” and “AC Area,” each also had a unique major peak. “HC Length” displayed a significant, yet transgressive QTL on chromosome 6 (LOD = 5.36) and “AC Area” displayed a significant QTL on chromosome 12 (LOD = 6.11).

### Candidate Genes for Autism-relevant Traits and AgCC in the BTBR Mouse

**Figure 6:**
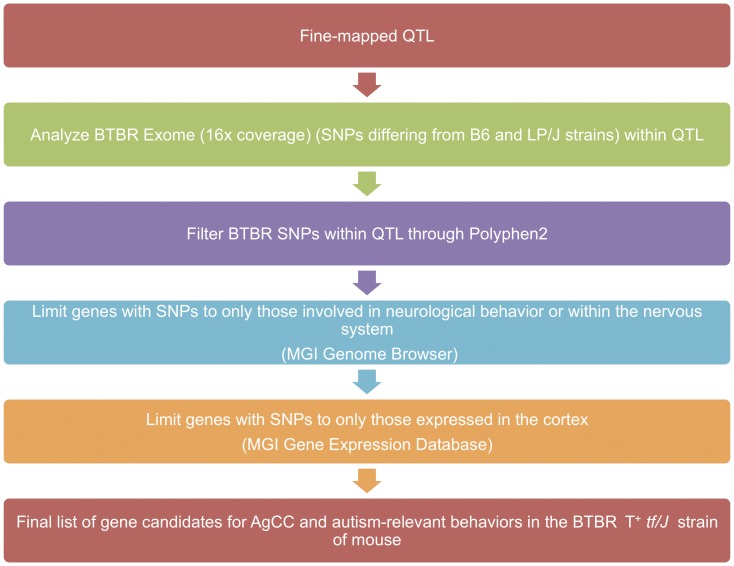
Gene candidate analysis flowchart. Following fine mapping, QTL meeting genome-wide significance of 5% were further analyzed to identify a list of gene candidates for future study. Sequencing data from the BTBR T^+^ *tf/J* strain was compared to known SNPs in the C57BL/6J and LP/J strains, and potential pathogenicity of BTBR-specific SNPs were determined (using Polyphen-2). Genes containing potentially pathogenic SNPs were filtered to only those involved in neurological behavior or within the nervous system, and then filtered again to limit genes to only those with cortical expression to develop the final list of gene candidates for AgCC and autism-relevant behaviors in the BTBR T^+^ *tf/J* strain of mouse.

We prioritized gene candidates based on novel and known potentially pathogenic variation within a gene, distance from the gene to the peak of the QTL, and hypothesis-driven spatial (cortex) and temporal (embryonic, early postnatal) transcript expression of the gene in mouse (Fig. 6). Table 2 summarizes these findings.

**Table 2:** List of Candidate Genes located within QTL Significant for Social Deficits and Callosal Phenotypes.

QTL	Chr	Candidate Gene(s)
Novel Mouse Sniff; Diff Sniff	3	Cyp7b1#
Novel Mouse Sniff; Diff Sniff	X	Vmn2r121, Olfr1320
Juvenile Nose to Nose	1	Pappa2, Prdx6, Rcsd1, Darc, Astn1, 4930455F23Rik, Fmn2, Igsf9
Juvenile Nose to Nose	9	Olfr160, Olfr887 Ankk1, Atm, Col6a4, Abhd14a, Celsr3, Scn11a, Xirp1* Rbm6, Camkv, Robo3#, Sorl1, Ube4a, Hspb2, Chrnb4, Unc13c, Tpbg, Acad11, Plxnb1, Crtap
Juvenile Push Crawl	10	Apaf1, Ankrd24, BC030307, Vezt
Juvenile Push Crawl	12	Nkx2-9, 1110034A24Rik, Syne2#
CC Length	4	Luzp1, 2610109H07Rik, Map3k6, Gpatch3, Sepn1, E2f2, Atp13a2#, Crocc, Spen, Fhad1, Tnfrsf1b, Tnfrsf8, Per3#
CC Area	4	Thrap3, Dcdc2b, Srsf4, Macf1, Ccdc28b#, Luzp1, 2610109H07Rik, Map3k6, Gpatch3, Sepn1, E2f2, Atp13a2#, Crocc, Spen, Fhad1, Tnfrsf1b, Tnfrsf8, Per3#
HC Length	4	Thrap3, Dcdc2b, Srsf4, Macf1, Ccdc28b#, Luzp1, 2610109H07Rik, Map3k6, Gpatch3, Sepn1, E2f2, Atp13a2#, Crocc, Spen, Fhad1, Tnfrsf1b, Tnfrsf8, Per3#
HC Length	6	Lmod3
AC Area	4	Luzp1, 2610109H07Rik, Map3k6, Gpatch3, Sepn1, E2f2, Atp13a2#, Crocc, Spen, Fhad1, Tnfrsf1b, Tnfrsf8, Per3#
AC Area	12	Greb1, Sox11

* Indicates gene has been previously implicated in ASD (SFARIgene; gene.sfari.org).

# Indicates gene family member has been previously implicated in ASD (SFARIgene; gene.sfari.org).

## Discussion

Over 250 genes have been implicated in the etiology of ASD (SFARI Gene; gene.sfari.org). The presence of an animal model of autism-relevant behaviors may allow for the identification of novel molecular and genetic contributors to this complex human disorder. The BTBR T+tf/J inbred mouse strain has been validated in multiple experiments in multiple laboratories as displaying behaviors that have face validity to the three main diagnostic categories of autism spectrum disorder, and recent studies using candidate pharmacologic treatments suggest that BTBR mice have shared biology with individuals with ASD [39], [40]. Our study is the first genetic mapping study of autism-relevant behaviors in the BTBR strain, allowing us to identify genetic contributors to these behaviors. Additionally, we have identified a highly significant locus responsible for callosal agenesis, as well as a number of loci contributing to the development of the two other forebrain commissures: hippocampal commissure and anterior commissure. This locus for callosal agenesis is particularly intriguing, as it has been previously implicated in AgCC in a 1998 study by Roy et al. [41] comparing the reduced callosum size of the NZB/BINJ strain to the C57BL/6By strain. Although we did not find the linkage to chromosome 1 noted by Roy et al. [41] in our study, we did find shared haplotypes between the BTBR T+tf/J and NZB/BINJ strains within this locus. If the shared haplotype can explain the observed genetic result, future studies examining the NZB/BINJ strain might help us identify the causative gene. The overlap of the peak in the two studies strongly implicates the chromosome 4 locus, and suggests that gene candidates within this locus might be important in determining the etiology of AgCC in inbred strains of mice.

**Figure 7:**
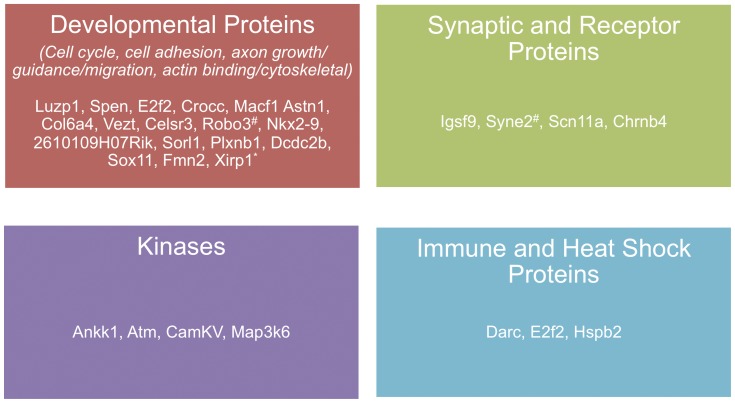
Categories of candidate genes for AgCC and autism-relevant phenotypes in the BTBR mouse. Candidate genes identified in the present study fall into four major protein categories: (1) Developmental proteins, involved in processes of cell cycle regulation, cell adhesion, axon growth, guidance, migration, actin-binding and cytoskeletal organization, (2) Synaptic and receptor proteins, (3) Kinases, and (4) Immune and heat shock proteins. ^*^Indicates gene has been previously implicated in ASD (SFARIgene; gene.sfari.org) ^#^Indicates gene family member has been previously implicated in ASD (SFARIgene; gene.sfari.org).

Furthermore, the linkage of loci to specific autism-relevant behaviors, in particular those related to abnormal juvenile and adult social behaviors, suggests that we may also be able to pinpoint causative genes within these loci. Analysis of the categories of the genes identified may help in this process. Figure 7 displays the categories of candidate genes identified in this study. These genes fall into four major categories: (1) developmental proteins, (2) synaptic and receptor proteins, (3) kinases, and (4) immune proteins. As expected, based on our method of gene identification, the majority of genes identified were involved with brain developmental processes, including those involved in cell cycle regulation, cell adhesion, axon growth/guidance, migration, and actin binding cytoskeletal elements, suggesting a role for these categories of genes in social behavior and callosal development.

In this first analysis, we have restricted our attention to genes that are interesting based on their known biology. In this way, by limiting gene candidates to those currently curated as being involved in “neurological behavior” or expressed “within the nervous system” within the MGI genome browser gene expression database, we may have inadvertently excluded novel gene candidates from our list that are unknown or more covert in their role in nervous system development, or that have yet to be curated. Furthermore, analysis of the proportion of variance explained by each QTL (Table 1), indicates that the genetic variance explained by the strongest detected QTL (CC Length) was 15.4%, and other QTLs explained even less variance, suggesting a more complex mechanism underlying agenesis of the corpus callosum and behaviors with face validity to human autism.

Of interest, we did not detect interactions between structural and behavioral loci, which was surprising, given the clinical literature indicating autism-like behaviors in patients with AgCC [16], [42]. This suggests that AgCC and autism-like phenotypes might be unrelated in the BTBR T+tf/J mouse strain, consistent with previous findings that there was no overt change in behavior after early postnatal transection of the corpus callosum [27]. Moreover, many of the cognitive deficits in executive function and higher order cognition, that are often impaired in patients with AgCC and with ASD, are difficult to model in rodents [16]. Nonetheless, many candidate genes involved in axonal guidance and migration were within behavioral loci, suggesting that there still may be a connection between aberrant white matter connectivity and ASD. Additionally, many of the genes identified within both structural and behavioral QTL were within families of genes curated in SFARIgene (gene.sfari.org), and the QTL were syntenic to regions containing CNVs previously associated with autism (https://gene.sfari.org/autdb/CNVHome.do), further suggesting their involvement in the etiology of aspects of ASD.

While the present study does not directly link specific gene variants to AgCC or ASD, the usage of an animal model of AgCC with autism-relevant behaviors has provided us with several significant QTL, each that contain a number of interesting candidate genes, to pursue in ongoing investigations of the genetic mechanisms of sociability and commissural connectivity.

## Supporting Information

Figure S1 **Social and repetitive behaviors in C57BL/6J (B6), BTBR T+tf/J (BTBR), and their F1 offspring (F1). Juvenile social interaction (A–D):** Each subject mouse was paired with an unfamiliar male B6 partner for a 10-min test in a Noldus Phenotyper arena. (A) BTBR and F1 engaged in significantly fewer direct nose-to-nose sniffs than B6. (B) There is a non-significant trend for BTBR and F1 to exhibit fewer front approaches than B6. (C) There is a non-significant trend for BTBR to exhibit fewer follows than B6. (D) BTBR and F1 engaged in significantly fewer push-crawls than B6. F1 scores were intermediate. **Adult social approach:** (E) B6 displayed normal sociability, spending more time in the chamber containing the novel mouse (blue bars) than in the chamber containing the novel object (white bars), BTBR spent equal time in both side chambers, indicating lower interest in a social partner. F1 scores were almost identical to those of BTBR. **F)** B6 spent significantly more time sniffing the novel mouse (blue bars) than the novel object (white bars), BTBR spent equal time sniffing the novel mouse and the novel object, indicating lower interest in a social partner. F1 scores were almost identical to those of BTBR. A–D, *p<.05 as compared to B6; E and F, *p<.01 novel mouse versus novel object. *B6 and BTBR data from A and C–F are adapted from Yang et al., 2009, European Journal of Neuroscience, Copyright (c) 2009, Blackwell Publishing, Ltd., with reuse and reprint permission via the Copyright Clearance Center (CCC).*
(TIF)
