# Supplementary figures and images for: Quantitative Trait Loci for Interhemispheric Commissure Development and Social Behaviors in the BTBR T+ tf/J Mouse Model of Autism
Source: PLoS One. 2013 Apr 15;8(4):e61829. doi: 10.1371/journal.pone.0061829 (PMC3626795; doi:10.1371/journal.pone.0061829)

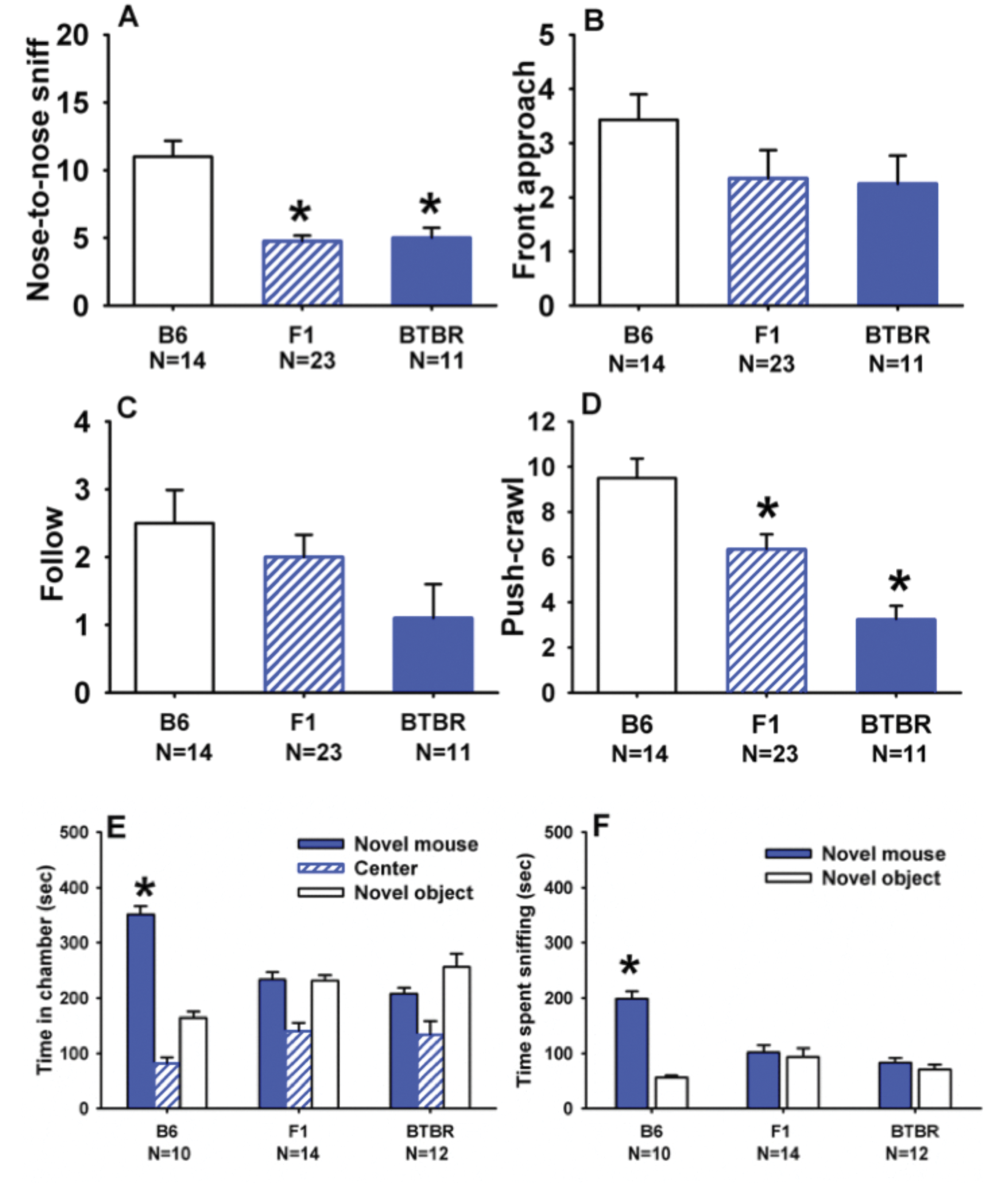

Supplement: Figure S1 — Social and repetitive behaviors in C57BL/6J (B6), BTBR T+tf/J (BTBR), and their F1 offspring (F1). Juvenile social interaction (A–D): Each subject mouse was paired with an unfamiliar male B6 partner for a 10-min test in a Noldus Phenotyper arena. (A) BTBR and F1 engaged in significantly fewer direct nose-to-nose sniffs than B6. (B) There is a non-significant trend for BTBR and F1 to exhibit fewer front approaches than B6. (C) There is a non-significant trend for BTBR to exhibit fewer follows than B6. (D) BTBR and F1 engaged in significantly fewer push-crawls than B6. F1 scores were intermediate. Adult social approach: (E) B6 displayed normal sociability, spending more time in the chamber containing the novel mouse (blue bars) than in the chamber containing the novel object (white bars), BTBR spent equal time in both side chambers, indicating lower interest in a social partner. F1 scores were almost identical to those of BTBR. F) B6 spent significantly more time sniffing the novel mouse (blue bars) than the novel object (white bars), BTBR spent equal time sniffing the novel mouse and the novel object, indicating lower interest in a social partner. F1 scores were almost identical to those of BTBR. A–D, *p<.05 as compared to B6; E and F, *p<.01 novel mouse versus novel object. B6 and BTBR data from A and C–F are adapted from Yang et al., 2009, European Journal of Neuroscience, Copyright (c) 2009, Blackwell Publishing, Ltd., with reuse and reprint permission via the Copyright Clearance Center (CCC). (TIF) [file pone.0061829.s001.tif]
